# *A1527G* variant of ABCA7 increases soluble and insoluble Aβ40‐42 in an animal model for late‐onset Alzheimer's disease.

**DOI:** 10.1002/alz70860_103215

**Published:** 2025-12-23

**Authors:** Cristian S Bernabe, Kevin P Kotredes, Ravi S Pandey, Gregory W Carter, Michael Sasner, Adrian L Oblak, Gareth R Howell, Bruce T. Lamb, Paul R Territo

**Affiliations:** ^1^ Stark Neurosciences Research Institute, Indianapolis, IN, USA; ^2^ The Jackson Laboratory, Bar Harbor, ME, USA; ^3^ Stark Neurosciences Research Institute, Indiana University School of Medicine, Indianapolis, IN, USA

## Abstract

**Background:**

The ATP binding cassette (ABC) subfamily A member 7 (*ABCA7*) gene was identified by Genome‐wide association studies (GWAS) as a risk factor for Alzheimer's disease (AD). *ABCA7* is part of the ABC1 subfamily and is expressed in brain cells including neurons, astrocytes, microglia, endothelial cells, and pericytes. Mounting evidence has linked ABCA7 variants to disrupted lipid metabolism, impaired microglia‐mediated phagocytosis, and altered amyloid deposition. However, the mechanisms by which variations in *ABCA7* increase risk for AD are not known.

**Method:**

The IU/JAX/PITT MODEL‐AD Center identified the *A1527G* variant in *ABCA7* (ABCA7*A1527G) as a putative LOAD risk factor. CRISPR/CAS9 was first used to introduce Abca7*A1527G variant to B6.APOE4.Trem2*R47H (LOAD1) mice to assess the transcriptional profiling on brain hemispheres from different ages. The Abca7*A1527G was then incorporated into B6.APOE4.Trem2*R47H.hAb (LOAD2) mice to further evaluate its contribution to LOAD. Female and male LOAD2.Abca7*A1527G and LOAD2 mice were characterized at 4, 12, and 24 months using the following phenotyping pipeline: behavior, PET/CT, multi‐omics, fluid biomarkers, neuropathology, and targeted protein analysis.

**Result:**

While no aging cognitive deficit was observed in LOAD2.Abca7*A1527G, PET/CT imaging revealed significant sex‐ and region‐dependent increases in brain glycolysis, accompanied by reduced tissue perfusion. This resulted in a progressive age‐related uncoupled phenotypes between 4‐12 and 4‐24 months. These changes were supported by brain transcriptional profiling showing Abca7*A1527G‐associated alterations in granulocyte/neutrophil migration, and insulin receptor signaling. Consistent with the uncoupled phenotype, while the addition of *A1527G* variant increased the plasma levels of IL6, IL10, and TNFα, brain levels of IL4, IL12, TNFα, and CXCL1 were decreased, and IL2 and IL10 were elevated independent of age. Interestingly, older ABCA7 cohorts had elevated soluble and insoluble Aβ40 and Aβ42 in both plasma and brain samples. Histological assessment of plaque deposition and microglia staining in 24‐month‐cohorts and targeted protein analysis are ongoing to determine the etiology of Aβ deposition.

**Conclusion:**

Data collected to date support a model whereby variations in *ABCA7* exert risk for AD through interactions between cerebrovasculature, microglia, Aβ deposition, and peripheral immune cells.

